# Do Temporomandibular Disorder Patients with Joint Pain Exhibit Forward Head Posture? A Cephalometric Study

**DOI:** 10.1155/2023/7363412

**Published:** 2023-02-02

**Authors:** Chu-Qiao Xiao, Yi-Dan Wan, Ya-Qi Li, Zhe-Bin Yan, Qiao-Yu Cheng, Pei-Di Fan, Yi Huang, Xiao-Yi Wang, Xin Xiong

**Affiliations:** ^1^National Clinical Research Center for Oral Diseases, State Key Laboratory of Oral Diseases, Department of Head and Neck Oncology Surgery, West China Hospital of Stomatology, Sichuan University, Chengdu, Sichuan, China; ^2^National Clinical Research Center for Oral Diseases, State Key Laboratory of Oral Diseases, Department of Orthodontics, West China Hospital of Stomatology, Sichuan University, Chengdu, Sichuan, China; ^3^Department of Nursing, West China Hospital of Stomatology, Sichuan University, Chengdu, Sichuan, China; ^4^Institute for Research of Children, Youth and Family, Masaryk University, Brno, Czech Republic

## Abstract

**Purpose:**

To evaluate head and cervical posture in individuals with or without temporomandibular disorders (TMDs) and to assess the correlations between pain, severity of symptoms, and posture.

**Methods:**

A total of 384 patients (129 males and 255 females) was included. The Fonseca Anamnestic Index (FAI) was used to assess the severity and prevalence of TMD and the presence of temporomandibular joint (TMJ) pain. Patients were divided into three groups: the TMD-free group, TMD without TMJ pain group, and TMD with TMJ pain group. Subsequently, the patients with TMJ pain were further divided into mild TMD and moderate/severe TMD groups. Nine parameters were traced on cephalograms to characterize the head and cervical posture.

**Results:**

TMD patients with TMJ pain showed increased forward head posture (FHP) than patients without TMJ pain and TMD-free subjects. No significant difference was observed between the TMD patients without TMJ pain and TMD-free subjects. In the TMD patients with the TMJ pain group, the moderate/severe TMD patients demonstrated increased FHP compared to mild TMD patients. TMD patients with joint pain had greater CVT/RL (*B* = 3.099), OPT/RL (*B* = 2.117), and NSL/C2' (*B* = 4.646) than the patients without joint pain after adjusting for confounding variables (*P* < 0.05).

**Conclusion:**

TMD patients with TMJ pain showed increased FHP compared to other groups, and FHP became more significant as TMD severity increased in male patients, indicating the FHP might play an important role in the development of TMJ pain. In the clinical assessment of TMD, the patients' abnormal head and cervical posture might be considered.

## 1. Introduction

Temporomandibular disorders (TMDs), with a reporting prevalence between 28% and 88%, are common in young people aged 20 to 40 years and in female [1]. TMD is a comprehensive disease involving joints, muscles, and nerves, which is mainly characterized by the restriction, deviation, and deflection of mandibular movement, clicking sound of temporomandibular joint (TMJ), and pain in masticatory muscles, anterior ear, and the TMJ region [2–4].

TMD might be caused by a variety of factors, including trauma, mental pressure, mandibular dysfunction, and malocclusion [5]. Among those, abnormal head and cervical posture has been reported to be associated with TMDs. Studies found that TMD patients had a higher cervical anteversion and an increased craniocervical angle [6]. Significant increases in craniovertebral, odontoid plane, and individual vertebral angles were also noted in TMD patients [7], while others did not identify association between head and cervical posture and TMD [8, 9]. Therefore, the relationship between the head and cervical posture and TMD remains controversial and needs further investigation.

Based on the biopsychosocial model, diagnostic criteria for temporomandibular disorders (DC/TMD) enable researchers to understand the relationship between physical and psychological factors and the development of TMD cases, which is a commonly used method for the diagnosis of TMD at present [10]. However, the use of DC/TMD is time-consuming and impractical in TMD fast screening. Currently, a number of simplified screening questionnaires for TMD have been proposed, and one of them is the Fonseca Anamnestic Index (FAI). FAI composed of only 10 questions has been widely used in many studies for its convenience and ease of use, which can quickly screen TMD patients in a large population [11, 12]. The FAI scale has been shown to be consistent with other tools for diagnosing TMD like Helkimo Index and the American Academy of Orofacial Pain questionnaire [13]. Zhang et al. [14] used DC/TMD as a criterion measure, and the Chinese version of the FAI has demonstrated good sensitivity (0.959) and specificity (0.719) and can be used to screen for TMD in Chinese population. In addition, FAI could compensate for the inability of DC/TMD to quantify TMD severity, allowing the researchers to evaluate patients based on the severity of TMD symptoms [11, 14].

Although TMD-related pain is not a life-threatening problem, it can affect oral health-related quality of life, and symptoms might transform into a chronic state and be difficult to control [15]. Like many other chronic pain syndromes, the biological mechanism of pain caused by TMD needs to be further studied. Using the FAI scale to diagnose TMD and identify pain in the TMJ area, our previous study has found that patients with TMJ pain had specific craniofacial features [16]. Nevertheless, the investigation about the relationship between head and cervical posture and TMJ pain is limited. The purpose of this study is to evaluate the head and cervical posture in patients with or without TMD, and furtherly explore the relationships between TMJ pain, severity of TMD, and the head and cervical posture. The null hypothesis is that there is no significant difference in the head and cervical posture between the TMD patients with and without TMJ pain.

## 2. Materials and Methods

### 2.1. Subjects

This study was conducted in accordance with the Helsinki Declaration and approved by the Ethics Committee of the West China Hospital of Stomatology in Sichuan University (Approval no. WCHSIRB-D-2021-431). After obtaining oral informed consent before the procedure, the participants provided written informed consent when filling the questionnaires. Parents or legal guardians of juvenile patients provided oral informed consent and written informed consent as well.

Patients visiting the Department of Orthodontics in the West China Hospital of Stomatology in Sichuan University from August to December in 2021 were consecutively included in this study. The patients were requested to fill out a questionnaire including demographic information and the FAI scale and were asked orally if they had a clinical history related to the exclusion criteria. Then, the lateral cephalograms of the patients performed at the Department of Medical Imaging in our hospital were collected. The inclusion criteria were as follows: (1) patients participating in this study voluntarily; (2) patients visiting our department for the first time; (3) patients filling out the questionnaire clearly; (4) patients aged 12 years or above; and (5) patients with clear cephalogram and in natural head position. The exclusion criteria were as follows: (1) patients with history of orthodontic or orthognathic treatment; (2) patients with head and neck trauma or tumour; (3) patients with congenital deformities of the head and the neck, such as cleft lip; (4) patients with systemic diseases, such as rheumatoid arthritis; (5) patients with severe dental, periodontal, and oral mucosal diseases; (6) patients with psychological disorders; (7) patients with the history of TMD treatment; (8) patients with the history of treatment for cervical; (9) patients with primary headaches; and (10) patients with severe malocclusion and craniofacial abnormalities.

### 2.2. Questionnaire

#### 2.2.1. Demographic Information

The demographic information of the patients included name, age, gender, educational level (“senior high school or lower”, “university,” or “graduate school or higher”), residence (“urban” or “rural”), and family per capita monthly income (“<3000 yuan,” “3000–6000 yuan,” or “>6000 yuan”).

#### 2.2.2. TMD and TMJ Pain Assessment

The FAI scale was used to assess the presence and severity of TMD in each patient [14, 17]. As described in our previous study [16], the total score of 10 questions reflected the presence and severity of TMD. Patients with a score of 0–15 were considered TMD-free patients, while patients with a score of 20 or higher were considered TMD patients. TMD patients could be further categorized based on TMD severity: a score of 20–40 = mild TMD, a score of 45–65 = moderate TMD, and a score of 70–100 = severe TMD [18].

The TMJ pain assessment was performed according to our previously reported method [16]. For TMD patients, if the answer of item 6 in FAI “Do you have ear pain or pain in the TMJ area?” was “sometimes” or “yes,” the patient was considered to be a TMD patient with TMJ pain. If the question was answered with a “no,” the patient was considered to be a TMD patient without TMJ pain.

### 2.3. Cephalometric Analysis

**Figure 1:**
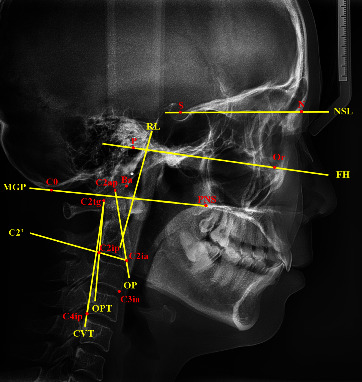
Cephalometric reference points and lines used in this study.

The lateral cephalograms of the patients were performed at the Department of Medical Imaging. The patients were required to maintain the natural head position with the mandible in the maximum intercuspal position, remain still, and not to swallow [19]. The cephalograms were collected from the database in the Department of Medical Imaging. Uceph software (version780, Yacent, Chengdu, Sichuan, China) was used for cephalometric analysis.  Figure 1 and Supplement Table 1 show the cephalometric reference points and lines used in this study.  Table 1 shows the 9 head and cervical posture parameters, including 2 linear measurements and 7 angular measurements [9, 20, 21].

Two blinded researchers performed the cephalometric analysis, and the intra- and interobserver reliability on cephalogram tracing was tested as previous described [16, 22]. No statistical differences between the two measurements of each researcher and between the measurements of two researchers were observed, and all intraclass correlation coefficients were >0.80 [23, 24].

### 2.4. Statistical Analysis

The sample size was computed by using G*∗*power (version 3.1.9, Germany). Kang [25] reported that the mean value for OPT/CVT in patients with painful TMD was −12.8° ± 1.3° compared with −11.7° ± 1.2° in the control group. Based on the previous cross-sectional studies, the prevalence of TMD was reported to be about 50% among the orthodontic patients [16], and we assumed that half of these patients have painful TMD. Keeping the power of the study as 90% and *α* as 0.05, the estimated minimum sample size was 108 subjects (54 in TMD-free group, 27 in TMD without TMJ pain group, and 27 in TMD with TMJ pain group) for this study. Considering that it may be beneficial to analyse the genders separately, a total of 384 subjects were included in this study.

All statistical analysis was conducted using IBM SPSS Statistics (version 20.0, IBM Corp, Armonk, NY, USA). *P* < 0.05 was considered to have a statistically significant difference. The quantitative data were expressed as mean and standard deviation, and the qualitative data were expressed by quantity and frequency. The Shapiro–Wilk test was used to judge the normality of data distribution. In order to compare the quantitative data of the patients in each group, independent sample *t*-test and one-way analysis of variance (ANOVA) were used when the data showed a normal distribution, and the Student-Newman-Keuls hoc test was used after ANOVA. The Mann–Whitney U-test or the Kruskal–Wallis H-test was used when the data did not show a normal distribution. In order to compare the qualitative data of the patients in each group, a chi-squared test was used. Spearman correlation analysis was used to correlate the FAI score and head and cervical posture parameters. Correlations were interpreted as follows: weak correlation, *r* < 0.30 and moderate or strong correlation, *r* >0.30. Also, the results of correlation analysis were visualized by scatterplots.

Multivariate linear regression was used to explore the correlation between TMJ pain in TMD patients and head and cervical posture parameters. The independent variable was TMJ pain, with “TMD patients without TMJ pain” as the reference group. The dependent variables were all head and cervical posture parameters. The other covariables including gender, age, educational level, residence, and family per capita monthly income were adjusted in the regression model.

## 3. Results

A total of 384 patients were included in this study, including 169 TMD-free patients (44.01%), 147 TMD patients without TMJ pain (38.28%), and 68 TMD patients with TMJ pain (17.71%). In terms of demographic characteristics, no significant difference in gender distribution was noted among the three groups (*P*=0.265). TMD patients without TMJ pain were significantly older than TMD-free patients, with no difference in the age between the TMD patients with TMJ pain and the other groups (*P* < 0.001). Concerning the educational level, the proportion of senior high school or lower in the TMD patients with TMJ pain was significantly higher than that in the other two groups (*P* < 0.001). No significant difference in residence and family per capita monthly income was observed among the three groups (*P* > 0.05). In the FAI survey, the score of FAI in the TMD group with TMJ pain was significantly higher than that in the other two groups (*P* < 0.001), and the proportion of moderate/severe TMD patients was significantly higher than that in the TMD group without TMJ pain (*P* < 0.001) (Table 2).

In all the male patients, the CVT/FH of TMD patients with TMJ pain was significantly larger than those of the TMD patients without TMJ pain and TMD-free patients (*P*=0.033). In all the female patients, the CVT/RL and NSL/C2' of TMD patients with TMJ pain were significantly larger than those of the other two groups (*P* < 0.05) (Table 3).

In adult population, no significant difference in the gender distribution among the three groups was noticed (*P*=0.276). In adult male patients, the CVT/RL, OPT/RL, and NSL/C2' of the TMD patients with TMJ pain were significantly larger than those of the other two groups (*P* < 0.05). In adult female patients, the CVT/RL and NSL/C2' of the TMD patients with TMJ pain were significantly larger than those of the other two groups (*P* < 0.05) (Table 4). Meanwhile, no significant differences in head and neck posture parameters were observed in the minor population (*P* > 0.05) (Supplement Table 2).

In the TMD patients with TMJ pain, stratified analysis based on the severity of TMD was performed (Table 5). No significant difference in gender distribution between mild TMD patients and moderate/severe TMD patients with TMJ pain existed (*P*=0.209). Among male, the craniocervical angle of moderate/severe TMD patients with TMJ pain was significantly smaller than that of the mild TMD patients, and the CVT/NSL, CVT/RL, OPT/NSL, and OPT/RL were significantly larger than those of the mild TMD patients (*P* < 0.05). However, no significant differences in head and neck posture parameters were observed between the two groups in female (*P* > 0.05).

**Figure 2:**
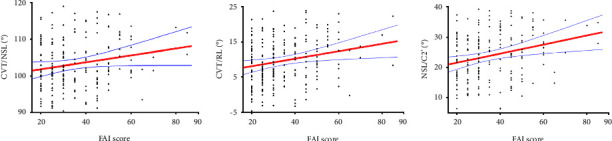
Scatterplot for the correlations between the FAI score and (a) CVT/NSL, (b) CVT/RL, and (c) NSL/C2' in overall patients. Scatterplot is fitted with the regression line (red line). The blue bands represent the 95% confidence interval.

In the overall study sample, the FAI score was weakly and positively correlated with CVT/NSL (*r* = 0.162, *P*=0.018), CVT/RL (*r* = 0.208, *P*=0.002), and NSL/C2' (*r* = 0.233, *P*=0.001). For male and female TMD patients, the FAI score was also weakly and positively correlated with several head and cervical posture parameters (*P* < 0.05) (Table 6). The correlations between the FAI score and CVT/NSL, CVT/RL, and NSL/C2' in overall patients were demonstrated using scatter plots (Figure 2).

The nonadjusted model showed that C2ap-C4ip, CVT/RL, OPT/RL, and NSL/C2' were positively correlated with the possibility of presenting TMJ pain in multivariate linear regression analysis. After adjustment for possible confounding factors, C2ap-C4ip became insignificant, and the possibility of presenting TMJ pain in the TMD patients were correlated with three head and neck posture parameters, including CVT/RL (*B* = 3.099, 95% CI: 1.172∼5.026, and *P*=0.002), OPT/RL (*B* = 2.117, 95% CI: 0.002∼4.232, and *P*=0.048), and NSL/C2' (*B* = 4.646, 95% CI: 2.209∼7.083, and *P* < 0.001) (Table 7).

## 4. Discussion

The main finding of this study was that there were significant differences in head and cervical posture between the TMD patients with and without TMJ pain, and the patients with TMJ pain have a significant trend of forward head posture (FHP). In the multivariate linear regression analysis, after adjustment for potential confounders, the increased in CVT/RL, OPT/RL, and NSL/C2' were independently associated with the TMJ pain risk. Thus, the null hypothesis was rejected.

The CVT/FH of male patients with TMJ pain and the CVT/RL and NSL/C2' of female patients with TMJ pain were significantly larger than those of the patients in other groups. Cephalometric results showed that the TMD patients with TMJ pain of both genders had a forward inclination of the upper segment of the cervical column and cervical hyperflexion. We then analysed head and cervical posture in adult patients. Adult TMD patients with TMJ pain for both genders also showed increased FHP when compared to TMD-free patients and TMD patients without TMJ pain. After adjusting for potential confounding factors, increased in CVT/RL, OPT/RL, and NSL/C2' remained significant risk factors for TMJ pain in the TMD patients. It demonstrated that the TMD patients with TMJ pain exhibited more pronounced FHP. Also, the increased in the length of the upper segment of the cervical column (C2ap-C4ip) might also be associated with the possibility of presenting TMJ pain. The significance was not statistical, but it was very close to the *P*=0.05 threshold.

In this study, the TMD patients with TMJ pain showed a significant FHP compared to the TMD-free patients and the TMD patients without TMJ pain. It is worth noting that there was no significant difference in head and cervical posture between the latter two groups. Therefore, a hypothesis was developed that a close relationship may exist between the FHP and the specific type of TMD with TMJ pain.

Previous studies have found that abnormal head and cervical posture was significantly associated with head and neck pain [26, 27]. This might be attributed to the complex anatomical structure and muscle connection between the functional unit formed by the cranium, mandible, and cervical spine, i.e., the craniocervical-mandibular system [28, 29]. From an anatomical point of view, when the FHP occurs, the postural misalignment could lengthen the anterior cervical muscles as well as shorten the posterior cervical muscles, and the increased pressure is exerted on the cervical facet joints and neck muscles [30, 31]. At the same time, a greater electromyographic activity in the temporal and masseter muscles could be observed in the TMD subjects [32, 33]. Increased pain sensitivity in the neck muscles and masticatory muscles in patients with TMD has also been detected [34–36]. Researchers reported that changes in cervical curvature could influence the muscle tension of the neck, subsequently affect the mandible movement and the muscle function in the TMJ area and eventually induce TMD [37, 38]. As for TMJ pain and headache related to TMD, they were also found to be associated with the dysfunction of the masticatory muscles [39, 40]. In conclusion, there is a tight relationship among head and cervical posture, muscle function, and TMD-related pain. It is very likely that FHP contributes to the development of TMJ pain, but the direct causal relationship between the two has not been determined because of the missing longitudinal studies.

Similarly, the FHP was found in patients with back pain, migraine, and chronic headache. Researchers also suggested that excessive stretch of muscles, soft tissues, and capsular ligaments beyond biological limitations from FHP may reduce the threshold of pain sensation in nerve endings and irritate proprioceptors in the joint capsules, which may contribute to these pain symptoms [26, 41–44].

Meanwhile, a severe FHP might not simply be a matter of the presence or absence of TMJ pain. We then further explored the relationship between TMD severity and head and cervical posture in the TMD patients with TMJ pain. Limited by the sample size, we had to combine patients with moderate and severe TMD into one group for statistical analysis, which inevitably overlooked some detailed information. We observed that the FHP appeared to become more significant in male patients with moderate/severe TMD than patients with mild TMD. Similarly, TMD severity in the TMD patients was weakly positively correlated with FHP in correlation analysis for both males and females. This finding suggested a possible “dose effect” could exist between the FHP and the signs and symptoms of TMD, and this requires further investigation based on a large sample size. Few studies have focused on the relationship between the head and cervical posture and TMD severity or the frequency of TMD-related symptoms, and the findings observed in this study in the TMD patients may provide some useful insights for subsequent research studies.

TMJ region pain and ear pain are common complaints in TMD patients [45]. To the best of our knowledge, there are few studies evaluating head and cervical posture in the TMD patients with TMJ pain. In this study, the findings of the specific head and cervical posture in these patients could contribute to our understanding of the underlying mechanisms of the development of TMD-related TMJ pain. So far, noninvasive, minimally invasive, and invasive treatments for TMJ pain usually focus on local pain conditions [46], while less attention has been given to the role of the head and cervical spine as a functional unit on the development of joint pain. In the clinical treatment for TMJ pain in the TMD patients, the effect of abnormal head and cervical posture might be considered. Although the previous studies have found that cognitive behavioral treatment [47, 48] and posture training [49, 50] can significantly relieve TMD symptoms including pain, treatments vary greatly among these studies, and the role of psychological factors and conventional treatments cannot be adequately adjusted [51]. This study identified organic changes in the head and neck posture in the TMD patients with joint pain, which provided the basic evidence of physiotherapy for pain-related TMD. In addition, the examination and intervention of abnormal head and neck postures are rarely included in the routine clinical practice. When treating TMD patients with main complaints of pain, it is necessary for physicians to pay attention to the relative position of their head and shoulders and to provide necessary explicit reminders and suggestions for posture training.

There are some limitations in this study. First, this study was a cross-sectional study and cannot establish a causal relationship between TMJ pain and head and cervical posture, and longitudinal investigations are necessary in the future. Second, this study only stratified patients based on the presence or absence of TMJ pain and did not quantify pain duration and pain intensity. Third, this study did not strictly distinguish between adults and adolescents. Adolescents at different growth stages may have specific cervical spine morphologies and head and neck posture characteristics due to their growth potential. Since this study included only a small number of adolescent TMD patients, the analysis of adolescent subjects alone may not meet the sample size requirement. It would be meaningful to increase the sample size of adolescents and analyse differences in their head and neck postures in further studies. Fourth, this study only used FAI to assess the severity and prevalence of TMD in patients without further verification of the diagnosis, which may result in grouping error and mislead the final conclusion. The use of DC/TMD could perform an accurate diagnosis of TMD and more detailed clinical subtypes of TMD patients in the follow-up studies.

## 5. Conclusion

TMD patients with TMJ pain showed abnormal head and cervical posture and increased FHP compared with TMD-free patients and TMD patients without TMJ pain. In male patients experiencing TMJ pain, the FHP appeared to become more significant in moderate/severe TMD patients compared to mild TMD patients. The FHP may play an important role in the development of TMJ pain in TMD. In the clinical treatment for TMD-related TMJ pain, the effect of abnormal head and cervical posture might be considered. Longitudinal investigations are necessary to define the causal relationship between the FHP and TMJ pain in the TMD patients.

## Figures and Tables

**Table 1:** Cephalometric parameters used in this study.

Cephalometric parameters	Definition
Ba-C3ia (mm)	The distance between basion and the most inferior-anterior point on the body of the third cervical vertebra. Higher values of Ba-C3ai represent more severe FHP.

C2ap-C4ip (mm)	The distance between the apex of the odontoid process of the second cervical vertebra and the most inferior-posterior point on the body of the fourth cervical vertebra (C4ip). C2ap-C4ip represents the length of the upper segment of the cervical column.

Cranio cervical angle (°)	The posterior-inferior angle of the intersection of the McGregor's plane (MGP) and odontoid plane (OP). Higher values of cranio cervical angle represent a flexion position of the head and in an FHP position.

CVT/OPT (°)	The anterior-inferior angle between the posterior tangent to the odontoid process through C4ip (CVT) and the posterior tangent to the odontoid process through C2ip (OPT). Higher values of CVT/OPT represent a larger cervical curvature and more severe FHP.

CVT/FH (°)	The anterior-inferior angle between CVT and Frankfort horizontal line (FH). Higher values of CVT/FH represent more severe FHP.

CVT/NSL (°)	The anterior-inferior angle between a line that goes from the sella turcica to the nasion and the tangent that goes posterior to the odontoid process through the most posterior and inferior aspect of the fourth cervical vertebra body. Higher values of CVT/NSL represent more severe FHP.

CVT/RL (°)	The anterior-inferior angle between a tangent line to the posterior border of the mandibular ramus and the tangent that goes posterior to the odontoid process through the most posterior and inferior aspect of the fourth cervical vertebra body. Higher values of CVT/RL represent more severe FHP.

OPT/RL (°)	The anterior-inferior angle between a tangent line to the posterior border of the mandibular ramus and the tangent that goes posterior to the odontoid process through the most posterior and inferior aspect of the second cervical vertebra body. Higher values of OPT/RL represent more severe FHP.

NSL/C2' (°)	The anterior-superior angle between a line that goes from the sella turcica to the nasion and the tangent line to the inferior edge of the second cervical vertebra. Higher values of NSL/C2' represent increase in cervical forward flexion in the second cervical segment and more severe FHP.

**Table 2:** Demographic characteristics and the FAI survey results of TMD-free patients, TMD patients without TMJ pain, and TMD patients with TMJ pain.

		TMD-free	TMD without TMJ pain	TMD with TMJ pain	*P*
Number (*n* (%))		169 (44.01%)	147 (38.28%)	68 (17.71%)	

Gender (*n* (%))	Male	62 (48.06%)	42 (32.56%)	25 (19.38%)	0.265
	Female	107 (41.96%)	105 (41.18%)	43 (16.86%)	

Age	Mean ± SD	21.82 ± 8.09	25.02 ± 6.67	23.29 ± 6.74	<0.001^*∗*^
	Median (IQR)	20.08 (14.58, 27.71)^a^	24.83 (19.75, 29.02)^b^	22.71 (18.35, 27.03)^a,b^	

Educational level (*n* (%))	Senior high school or lower	25 (22.94%)^a^	19 (17.43%)^a,b^	65 (59.63%)^b^	<0.001^*∗*^
	Undergraduate	98 (44.14%)^a^	36 (16.22%)^a,b^	88 (39.64%)^b^	
	Graduate or higher	24 (45.28%)^a^	13 (24.53%)^a^	16 (30.19%)^a^	

Residence (*n* (%))	Urban	14 (35.90%)	9 (23.08%)	16 (41.02%)	0.677
	Rural	133 (38.55%)	59 (17.10%)	153 (44.35%)	

Family per capita monthly income (*n* (%))	<3000 yuan	15 (50.00%)	4 (13.33%)	11 (36.67%)	0.448
	3000–6000	53 (40.77%)	25 (19.23%)	52 (40.00%)	
	>6000 yuan	79 (35.27%)	39 (17.41%)	106 (47.32%)	

FAI scores	Mean ± SD	7.25 ± 5.69	29.90 ± 9.80	46.18 ± 14.82	<0.001^*∗*^
	Median (IQR)	5 (0, 15)^a^	25 (20, 35)^b^	45 (35, 55)^c^	

TMD severity (*n* (%))	Mild TMD	—	127 (82.47%)	27 (15.73%)	<0.001^*∗*^
	Moderate/severe TMD	—	20 (32.79%)	41 (67.21%)	

Notes: The chi-squared test and the Kruskal–Wallis H-test were used. Different superscript letters indicate significant differences, and ^*∗*^*P* < 0.05. TMD, temporomandibular disorders; TMJ, temporomandibular joint; FAI, the Fonseca Anamnestic Index; SD, standard deviation; IQR, interquartile range.

**Table 3:** Head and cervical posture parameters of TMD-free patients, TMD patients without TMJ pain, and TMD patients with TMJ pain.

Head and cervical posture parameters		TMD-free	TMD without TMJ pain	TMD with TMJ pain	*P*	Post hoc test
Number (*n*(%))	Male	62 (48.06%)	42 (32.56%)	25 (19.38%)	0.265	
	Female	107 (41.96%)	105 (41.18%)	43 (16.86%)		

Ba-C3ia (mm)	Male	60.78 ± 4.97	59.36 ± 4.59	61.15 ± 4.85	0.235	
	Female	54.32 ± 3.86	54.99 ± 3.61	55.36 ± 3.72	0.522	

C2ap-C4ip (mm)	Male	70.14 ± 5.59	68.69 ± 4.32	70.71 ± 5.67	0.080	
	Female	62.75 ± 4.65	63.65 ± 3.69	64.33 ± 4.28	0.084	

Craniocervical angle (°)	Male	101.88 ± 8.04	102.57 ± 7.83	99.41 ± 6.60	0.327	
	Female	103.58 ± 7.44	102.55 ± 8.44	102.13 ± 7.20	0.488	

CVT/OPT (°)	Male	3.75 ± 2.61	3.26 ± 3.41	3.06 ± 2.56	0.528	
	Female	4.41 ± 2.95	4.27 ± 2.74	4.32 ± 2.55	0.930	

CVT/FH (°)	Male	87.33 ± 9.05	86.18 ± 7.76	91.79 ± 9.05	0.033^*∗*^	3 > 2, 3 > 1
	Female	87.90 ± 7.26	87.85 ± 8.48	87.52 ± 7.72	0.964	

CVT/NSL (°)	Male	103.59 ± 7.30	103.85 ± 6.68	104.53 ± 6.04	0.800	
	Female	101.40 ± 6.93	102.31 ± 8.01	104.2 ± 8.58	0.121	

CVT/RL (°)	Male	11.25 ± 7.16	10.17 ± 5.86	12.58 ± 7.45	0.374	
	Female	8.14 ± 6.12	8.21 ± 6.96	11.33 ± 5.78	0.015^*∗*^	3 > 2, 3 > 1

OPT/RL (°)	Male	7.50 ± 7.65	6.91 ± 7.10	9.52 ± 7.75	0.373	
	Female	3.73 ± 6.89	4.04 ± 7.57	5.59 ± 6.28	0.337	

NSL/C2' (°)	Male	23.80 ± 9.11	22.02 ± 8.34	26.93 ± 6.9	0.076	
	Female	22.27 ± 9.13	22.57 ± 8.44	26.8 ± 8.51	0.007^*∗*^	3 > 2, 3 > 1

Notes: The chi-squared test, one-way analysis of variance, and the Kruskal–Wallis H-test were used. In the post hoc test column, numbers “1”, “2,” and “3” represent TMD-free, TMD without TMJ pain, and TMD with TMJ pain groups of the subjects, respectively, ^*∗*^*P* < 0.05. TMD, temporomandibular disorders; TMJ, temporomandibular joint.

**Table 4:** Head and cervical posture parameters of TMD-free patients, TMD patients without TMJ pain, and TMD patients with TMJ pain in the adult population.

Head and cervical posture parameters		TMD-free	TMD without TMJ pain	TMD with TMJ pain	*P*	Post hoc test
Number (*n* (%))	Male	41 (43.16%)	36 (37.89%)	18 (18.95%)	0.276	
	Female	68 (34.52%)	93 (47.21%)	36 (18.27%)		

Ba-C3ia (mm)	Male	61.66 ± 3.93	59.27 ± 4.81	61.62 ± 3.72	0.035^*∗*^	1 > 2
	Female	54.39 ± 3.76	54.87 ± 3.65	55.25 ± 3.85	0.510	

C2ap-C4ip (mm)	Male	71.46 ± 4.89	68.84 ± 4.60	71.91 ± 4.38	0.023^*∗*^	1 > 2, 3 > 2
	Female	62.98 ± 4.22	63.70 ± 3.82	64.34 ± 4.21	0.376	

Cranio cervical angle (°)	Male	102.24 ± 8.70	102.80 ± 7.01	98.01 ± 7.18	0.092	
	Female	103.76 ± 6.88	102.45 ± 8.42	101.55 ± 7.38	0.340	

CVT/OPT (°)	Male	3.81 ± 2.82	2.91 ± 3.37	2.36 ± 2.29	0.174	
	Female	4.31 ± 2.68	4.22 ± 2.63	4.24 ± 2.62	0.978	

CVT/FH (°)	Male	86.53 ± 9.12	86.50 ± 7.80	91.62 ± 10.28	0.095	
	Female	86.74 ± 6.29	87.93 ± 8.61	86.74 ± 8.05	0.561	

CVT/NSL (°)	Male	103.78 ± 7.00	103.87 ± 6.16	105.65 ± 6.38	0.436	
	Female	101.34 ± 6.51	102.40 ± 8.04	104.98 ± 8.97	0.065	

CVT/RL (°)	Male	11.10 ± 6.95	10.08 ± 5.51	14.67 ± 5.97	0.041^*∗*^	3 > 2, 3 > 1
	Female	8.12 ± 5.06	8.13 ± 6.49	11.94 ± 5.64	0.002^*∗*^	3 > 2, 3 > 1

OPT/RL (°)	Male	7.29 ± 7.56	7.16 ± 6.51	12.31 ± 5.70	0.021^*∗*^	3 > 2, 3 > 1
	Female	3.81 ± 5.59	4.02 ± 7.02	6.00 ± 6.35	0.214	

NSL/C2' (°)	Male	23.66 ± 8.24	21.91 ± 7.75	28.16 ± 7.52	0.027^*∗*^	3 > 2, 3 > 1
	Female	22.26 ± 8.11	22.75 ± 8.39	27.85 ± 8.50	0.002^*∗*^	3 > 2, 3 > 1

Notes: The chi-squared test, one-way analysis of variance, and the Kruskal–Wallis H-test were used. In the post hoc test column, numbers “1”, “2,” and “3” represent TMD-free, TMD without TMJ pain and TMD with TMJ pain groups of the subjects, respectively, ^*∗*^*P* < 0.05. TMD, temporomandibular disorders; TMJ, temporomandibular joint.

**Table 5:** Head and cervical posture parameters of mild TMD patients with TMJ pain and moderate/severe TMD patients with TMJ pain.

Head and cervical posture parameters		Mild TMD with TMJ pain	Moderate/severe TMD with TMJ pain	*P*
Number (*n*(%))	Male	12 (48.00%)	13 (52.00%)	0.209
	Female	15 (34.88%)	28 (65.12%)	

Ba-C3ia (mm)	Male	60.80 ± 6.09	61.47 ± 3.59	0.738
	Female	54.84 ± 4.17	55.65 ± 3.50	0.504

C2ap-C4ip (mm)	Male	69.94 ± 6.99	71.42 ± 4.27	1.000
	Female	63.63 ± 5.17	64.70 ± 3.77	0.486

Cranio cervical angle (°)	Male	102.49 ± 4.56	96.56 ± 7.05	0.020^*∗*^
	Female	102.87 ± 6.31	101.73 ± 7.71	0.625

CVT/OPT (°)	Male	3.56 ± 2.30	2.60 ± 2.79	0.359
	Female	4.37 ± 2.47	4.29 ± 2.64	0.916

CVT/FH (°)	Male	90.75 ± 5.68	92.75 ± 11.50	0.584
	Female	87.59 ± 6.65	87.48 ± 8.36	0.967

CVT/NSL (°)	Male	101.75 ± 5.47	107.10 ± 5.53	0.019^*∗*^
	Female	103.45 ± 8.32	104.61 ± 8.83	0.678

CVT/RL (°)	Male	8.65 ± 7.15	16.21 ± 5.87	0.008^*∗*^
	Female	10.75 ± 5.34	11.64 ± 6.07	0.635

OPT/RL (°)	Male	5.09 ± 7.44	13.61 ± 5.62	0.004^*∗*^
	Female	5.57 ± 5.00	5.60 ± 6.95	0.989

NSL/C2' (°)	Male	24.4 ± 7.22	29.27 ± 5.94	0.077
	Female	25.06 ± 9.85	27.73 ± 7.73	0.386

Notes: The chi-squared test, the independent sample *t*-test, and the Mann–Whitney test were used, ^*∗*^*P* < 0.05. TMD, temporomandibular disorders; TMJ, temporomandibular joint.

**Table 6:** Correlation between the FAI score and head and cervical posture parameters in TMD patients.

Head and cervical posture parameters	Overall (*N* = 215)		Male (*N* = 67)		Female (*N* = 148)	
	*P*	*r*	*P*	*r*	*P*	*r*
Ba-C3ia (mm)	0.216	0.085	0.038	0.254^*∗*^	0.952	0.005
C2ap-C4ip (mm)	0.423	0.055	0.045	0.246^*∗*^	0.872	−0.013
Cranio cervical angle (°)	0.110	−0.109	0.127	−0.188	0.343	−0.079
CVT/OPT (°)	0.647	0.031	0.645	0.057	0.711	0.031
CVT/FH (°)	0.602	0.036	0.065	0.227	0.547	−0.050
CVT/NSL (°)	0.018	0.162^*∗*^	0.308	0.126	0.033	0.175^*∗*^
CVT/RL (°)	0.002	0.208^*∗*^	0.042	0.250^*∗*^	0.010	0.212^*∗*^
OPT/RL (°)	0.051	0.134	0.127	0.188	0.136	0.123
NSL/C2' (°)	0.001	0.233^*∗*^	0.052	0.238	0.005	0.228^*∗*^

Notes: Spearman correlation analysis is used. ^*∗*^*P* < 0.05.

**Table 7:** Multivariate linear regression analysis between the possibility of presenting TMJ pain and head and cervical posture parameters, adjusted for gender, age, educational level, residence, and family per capita monthly income.

Head and cervical posture parameters	Nonadjusted				Adjusted model			
	*B*	*P*	95% CI		*B*	*P*	95% CI	
Ba-C3ia (mm)	1.249	0.064	−0.075	2.573				
C2ap-C4ip (mm)	1.579	0.029^*∗*^	0.161	2.997	1.092	0.083	−0.146	2.329
Cranio cervical angle (°)	−1.426	0.219	−3.707	0.855				
CVT/OPT (°)	−0.125	0.766	−0.953	0.703				
CVT/FH (°)	1.719	0.161	−0.690	4.128				
CVT/NSL (°)	1.579	0.162	−0.639	3.797				
CVT/RL (°)	3.018	0.002^*∗*^	1.106	4.930	3.099	0.002^*∗*^	1.172	5.026
OPT/RL (°)	2.179	0.046^*∗*^	0.044	4.315	2.117	0.048^*∗*^	0.002	4.232
NSL/C2' (°)	4.432	0.000^*∗*^	2.051	6.813	4.646	0.000^*∗*^	2.209	7.083

Notes: Adjusted model adjusts for gender, age, educational level, residence, and family per capita monthly income. ^*∗*^*P* < 0.05. TMD, temporomandibular disorder; TMJ, temporomandibular joint; CI, confidence interval.

## Data Availability

The data used to support the findings of this study are available from the corresponding author upon request.

## Supplementary Materials

Supplementary Materials Supplement Table 1: cephalometric reference points and lines used in this study and their definition. Supplement Table 2: head and cervical posture parameters of TMD-free patients, TMD patients without TMJ pain, and TMD patients with TMJ pain in the minor population.
