# Supplementary material for: Do Temporomandibular Disorder Patients with Joint Pain Exhibit Forward Head Posture? A Cephalometric Study
Source: Pain Res Manag. 2023 Feb 2;2023:7363412. doi: 10.1155/2023/7363412 (PMC9911253; doi:10.1155/2023/7363412)
Supplement: Supplementary Materials — Supplement Table 1: cephalometric reference points and lines used in this study and their definition. Supplement Table 2: head and cervical posture parameters of TMD-free patients, TMD patients without TMJ pain, and TMD patients with TMJ pain in the minor population. [file 7363412.f1.docx]

**Supplement Table 1** Cephalometric reference points and lines used in this study and their definition

| **Cephalometric reference points** | **Definition** |
| --- | --- |
| C0 | The base of the occiput. |
| C2ap | The apex of the odontoid process of the second cervical vertebra. |
| C2ia | The most inferior-anterior point on the body of the second cervical vertebra. |
| C2ip | The most inferior-posterior point on the body of the second cervical vertebra. |
| C2tg | The tangent point of odontoid process tangent (OPT) on the odontoid process of the second cervical vertebra. |
| C3ia | The most inferior-anterior point on the body of the third cervical vertebra. |
| C4ip | The most inferior-posterior point on the body of the fourth cervical vertebra. |
| N | Nasion. |
| S | Sella. |
| P | Porion. |
| Or | Orbitale. |
| Ba | Basion. |
| PNS | Posterior nasal spine. |
| **Cephalometric reference lines** |  |
| MGP | McGregor's Plane, the line through C0 and PNS. |
| OP | Odontoid Plane, the line through C2ap and C2ia. |
| CVT | Cervical vertebrae tangent, the posterior tangent to the odontoid process through C4ip. |
| OPT | Odontoid process tangent, the posterior tangent to the odontoid process through C2ip. |
| NSL | Nasion-Sella line, the line through Nasion and Sella. |
| FH | Frankfort horizontal line, the line through Orbitale and Porion. |
| RL | Ramus line, tangent to the posterior border of the mandibular ramus. |
| C2’ | The line through C2ia and C2ip. |

**Supplement Table 2** Head and cervical posture parameters of TMD-free patients, TMD patients without TMJ pain and TMD patients with TMJ pain in the minor population

| **Head and cervical posture parameters** |  | **TMD-free** | **TMD without TMJ pain** | **TMD with TMJ pain** | ***P*** |
| --- | --- | --- | --- | --- | --- |
| Number (n (%)) | Male | 21 (61.76%) | 6 (17.65%) | 7 (20.59%) | 0.505 |
|  | Female | 41 (68.33%) | 12 (20.00%) | 7 (11.67%) |  |
| Ba-C3ia (mm) | Male | 59.06±6.30 | 59.92±3.19 | 59.93±7.25 | 0.924 |
|  | Female | 54.41±4.12 | 55.94±3.30 | 55.97±3.15 | 0.366 |
| C2ap-C4ip (mm) | Male | 67.55±6.08 | 67.8±1.90 | 67.63±7.66 | 0.996 |
|  | Female | 62.48±5.26 | 63.29±2.52 | 64.27±4.99 | 0.625 |
| Cranio cervical angle (°) | Male | 101.2±6.72 | 101.19±12.52 | 102.99±2.69 | 0.852 |
|  | Female | 103.25±8.20 | 103.3±8.94 | 105.13±5.69 | 0.850 |
| CVT/OPT (°) | Male | 3.62±2.21 | 5.37±3.13 | 4.87±2.45 | 0.226 |
|  | Female | 4.65±3.32 | 4.62±3.61 | 4.73±2.34 | 0.997 |
| CVT/FH (°) | Male | 88.91±8.92 | 84.25±7.91 | 92.24±5.31 | 0.229 |
|  | Female | 89.68±8.26 | 87.21±7.65 | 91.54±4.07 | 0.472 |
| CVT/NSL (°) | Male | 103.24±8.02 | 103.71±10.02 | 101.67±4.16 | 0.873 |
|  | Female | 101.64±7.53 | 101.54±8.05 | 100.21±4.81 | 0.893 |
| CVT/RL (°) | Male | 11.55±7.72 | 10.74±8.25 | 7.21±8.62 | 0.469 |
|  | Female | 8.23±7.52 | 8.84±10.24 | 8.21±5.88 | 0.973 |
| OPT/RL (°) | Male | 7.93±7.98 | 5.37±10.68 | 2.34±8.04 | 0.320 |
|  | Female | 3.58±8.58 | 4.22±11.42 | 3.48±5.90 | 0.975 |
| NSL/C2’ (°) | Male | 24.07±10.83 | 22.64±12.20 | 23.77±3.75 | 0.955 |
|  | Female | 22.58±10.64 | 21.18±9.01 | 21.36±6.70 | 0.891 |

**Notes:** Chi-squared test, one-way analysis of variance and Kruskal-Wallis H test were used.

**Abbreviations:** TMD: temporomandibular disorders, TMJ: temporomandibular joint.
